# Response to: Comment on “48-Week Outcome after Cessation of Nucleos(t)ide Analogue Treatment in Chronic Hepatitis B Patient and the Associated Factors with Relapse”

**DOI:** 10.1155/2019/2970510

**Published:** 2019-05-02

**Authors:** Wen-xiong Xu, Liang Peng

**Affiliations:** ^1^Department of Infectious Diseases, Third Affiliated Hospital of Sun Yat-sen University, Guangzhou 510630, Guangdong, China; ^2^Guangdong Key Laboratory of Liver Disease Research, Third Affiliated Hospital of Sun Yat-sen University, Guangzhou 510630, Guangdong, China

We appreciate the interest of Yafei Guo and colleagues [1] in our publication [2]. We agree with Guo et al. that nucleos(t)ide analogues (NAs) cessation can be recommended in chronic hepatitis B (CHB) patients who are relatively younger and have relatively low serum hepatitis B surface antigen (HBsAg) levels and that it may be more effective to add on interferon treatment as an immune modulation rather than NAs cessation for CHB patients with low-level HBsAg.

In our study, we recruited patients with undetected HBV DNA for a period of time before NAs cessation, which is described in the inclusion criteria, according to the guidelines of prevention and treatment of CHB from the Asian Pacific Association for the Study of the Liver (APASL) [3]. Although 39 of 62 patients experienced virologic relapse in our study, 14 patients changed to the nonrelapse group, with a relatively low level of HBV DNA. This may be a balance between the virus and host immunity, which results from immune recovery and self-control by the patients themselves [4]. Although the number of these patients is small, we think NAs cessation can be achieved in case of long-term follow-up.

In 10 patients with HBsAg clearance before NAs cessation, HBsAg clearance and undetectable HBV DNA are maintained throughout follow-up. These patients are “functionally cured”. So NAs cessation in patients with HBsAg clearance is achievable. For CHB patients with low-level HBsAg, NAs cessation can be recommend by a systematic review [5]. Switching to or adding on interferon alpha treatment can be another choice for those who have not contraindications and who can afford the treatment, as interferon alpha is an immune modulator. A study of NAs cessation and switching to interferon alpha treatment in CHB patients with low-level HBsAg is proceeding to achieve HBsAg seroconversion right now in our team. The results were presented at the 2018 AASLD Liver Meeting [6].

There are few parameters, such as HBsAg or pgRNA, for predicting relapse and NAs retreatment in CHB patients. Further study is needed.
